# Testing a proposed mathematical model of weight loss in women enrolled on a commercial weight-loss programme: the LighterLife study

**DOI:** 10.1017/jns.2024.85

**Published:** 2024-12-12

**Authors:** Aoife M. Egan, John F. Rayman, Adam L. Collins

**Affiliations:** 1 Faculty of Health & Medical Sciences, University of Surrey, Guildford, United Kingdom of Great Britain and Northern Ireland; 2 Department of Mathematics, University of Surrey, Guildford, United Kingdom of Great Britain and Northern Ireland

**Keywords:** adaptive thermogenesis, fat-free mass, mathematical modelling, weight loss

## Abstract

Weight loss results in obligatory reductions in energy expenditure (EE) due to loss of metabolically active fat-free mass (FFM). This is accompanied by adaptive reductions (i.e. adaptive thermogenesis) designed to restore energy balance while in an energy crisis. While the ‘3500-kcal rule’ is used to advise weight loss in clinical practice, the assumption that EE remains constant during energy restriction results in a large overestimation of weight loss. Thus, this work proposes a novel method of weight-loss prediction to more accurately account for the dynamic trajectory of EE. A mathematical model of weight loss was developed using ordinary differential equations relying on simple self-reported inputs of weight and energy intake to predict weight loss over a specified time. The model subdivides total daily EE into resting EE, physical activity EE, and diet-induced thermogenesis, modelling obligatory and adaptive changes in each compartment independently. The proposed model was tested and refined using commercial weight-loss data from participants enrolled on a very low-energy total-diet replacement programme (LighterLife UK, Essex). Mathematical modelling predicted post-intervention weight loss within 0.75% (1.07 kg) of that observed in females with overweight or obesity. Short-term weight loss was consistently underestimated, likely due to considerable FFM reductions reported on the onset of weight loss. The best model agreement was observed from 6 to 9 weeks where the predicted end-weight was within 0.35 kg of that observed. The proposed mathematical model simulated rapid weight loss with reasonable accuracy. Incorporated terms for energy partitioning and adaptive thermogenesis allow us to easily account for dynamic changes in EE, supporting the potential use of such a model in clinical practice.

## Introduction

The energy balance (EB) principle, that is, the first law of thermodynamics, states that energy can be neither created nor destroyed, but only transformed. The human body is considered an open system, where energy (kcal) is added in the form of food (‘calories in’) and transformed to combustion to produce heat (‘calories out’)^(1)^. Thus, EB represents the relationship between energy intake (EI) and energy expenditure (EE). Any imbalance between EI and EE results in a shift in energy stores and a subsequent change in weight as fat mass (FM) and fat-free mass (FFM).

The EB equation provides the basis of weight-loss strategies currently implemented in clinical practice. The ‘3500-kcal rule’ is a guidance used in clinical weight management that advises that a reduction of 500 kcal/d will result in 1 lb (∼0.5 kg) weight loss per week^(2)^. This approach relies on the assumption that FFM accounts for 25% of total weight loss with the remaining 75% lost as FM. This represents a ‘static model of weight loss’, where weight is lost at a fixed rate, decreasing in a linear manner during periods of dynamic weight loss^(3)^.

However, while the principle of EB appears straightforward, quantifying energy imbalance during periods of energy restriction is much more complex. The first inaccuracy of the 3500-kcal rule lies in the assumption that the proportion of weight lost as FM and FFM remains constant during the weight-loss phase^(2)^. In actuality, the fraction of weight lost as either component changes during the weight-loss phase ranges between 20–40% for FFM and 60–80% FM, respectively^(4)^, resulting in dynamic shifts in energy imbalance.

Second, while traditionally assumed to be independent variables, the components of EB, that is, EI and EE, are in fact functionally interdependent, with changes in one side of the equation corresponding with alterations in the other side^(5,6)^. The evidence is very clear that if you restrict calories, EE will decline simultaneously^(7)^. This typically occurs in two ways: (i) obligatory reductions in EE resulting from a loss of metabolic tissue and (ii) adaptive reductions in EE resulting from ‘adaptive thermogenesis’ (AT), defined as the underfeeding-associated fall in resting energy expenditure (REE) independent of changes in FFM and FM^(8,9)^.

Thus, a 500-kcal deficit per day does not directly translate into 1 lb (0.5 kg) weight loss as traditionally assumed, resulting in less-than-predicted weight loss^(10,11)^.

To account for such shifts in EB during the weight-loss phase, various mathematical models^(12)^ (also referred to as dynamic models) have been developed based on the EB principle, simulating the non-linear nature of weight loss by modelling the dynamic changes in EE resulting from FFM loss and AT. Such models vary in complexity depending on (i) the way EE is subdivided and (ii) the way body mass is compartmentalised. While accurate models do exist, they often require complex parameters that are unobtainable in a clinical setting, often limited to simple demographics (e.g. gender and age) and anthropometric measures (e.g. height, weight, waist circumference).

The aim of this study is to produce a working mathematical model of weight loss requiring only simple baseline parameters to more accurately describe the dynamic trajectory of weight loss over a given time. Subsequently, the model will be tested and refined using a large database of female clients enrolled on a commercial weight management programme (LighterLife, Essex, United Kingdom).

## Methods

The present study describes a mathematical model of weight loss developed based on estimates of the components of EB and how these change during periods of weight loss. Values used in the development of the model are determined based on existing observational weight-loss data.

### Model development

The proposed mathematical model uses baseline inputs of weight (kg), EI (kcal), and physical activity level (PAL) to predict weight loss over a specified time. The model subdivides total daily energy expenditure into resting energy expenditure (REE), physical activity energy expenditure (PAEE), and diet-induced thermogenesis (DIT), modelling each compartment independently. An energy conversion of 7700 kcal per kg is assumed to convert energy deficit to weight loss as described by Equation 1:

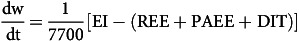




Equation 1: Simple equation for weight loss. 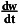 represents the rate of weight change over a given time. Weight loss is assumed as the discrepancy between EI and EE. EI is energy intake in kcal/d. Energy expenditure is subdivided into REE, PAEE, and DIT. REE is resting energy expenditure in kcal/d, PAEE is physical activity energy expenditure in kcal/d, and DIT is diet-induced thermogenesis in kcal/d. An energy conversion of 7700 kcal/kg is assumed to convert energy deficit to weight loss.

### Energy intake and energy expenditure

EI (kcal/d) is used as a model input and is assumed true to that reported. REE (kcal/d) is estimated using the Cunningham Equation based on FFM^(13)^. Baseline FFM% is estimated using gender-specific expressions developed by our group as described by Equation 2:

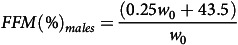




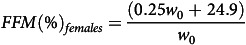




Equation 2: Predictive equations for starting FFM (%). A linear relationship between starting body weight and FFM (kg) was assumed where starting values for FFM of a lean male and female were obtained from the literature and excess body weight was assumed to be composed of 75% FM and 25% FFM. This value was divided by starting body weight, allowing us to predict baseline FFM (%). *FFM*, fat-free mass; *FM*, fat mass; *w*_*0*_, weight (kg).

PAL index^(14)^ is used to account for PAEE (kcal/d), where TEE is calculated as REE multiplied by PAL (TEE = REE x PAL). Values range from 1.2 (chair-bound/bed-bound) to 2.4 (strenuous work or highly active leisure) and are self-reported to most accurately reflect the individual’s lifestyle. DIT (kcal/d) is assumed a direct product of EI and is estimated using a coefficient of 10% in a lean population and 5% in an overweight/obese population^(15)^.

### Obligatory changes in energy expenditure

FFM is modelled as a function of weight loss. An exponential increase in FFM% is assumed on the onset of weight loss, persisting until an overall weight loss of 10% is achieved at which point FFM% stabilises approximately 10 percentage points higher than baseline. The predicted value for FFM% is multiplied by body weight at any given time to produce a predicted value for FFM (kg), as described by Equation 3:

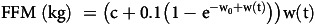




Equation 3: Original expression for FFM change.

*FFM*, fat-free mass; *c*, baseline fat-free mass (%); *w*_*0*_, starting weight; *w(t)*, weight at any given time.

### Adaptive changes in energy expenditure

AT is modelled as a function of weight loss. An exponential increase in AT is assumed during the weight-loss phase, persisting until a total weight loss of 10% is achieved, at which point AT stabilises at a value equivalent to 15% of REE^(16,17)^ as described by Equation 4:

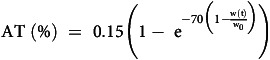




Equation 4: Expression for adaptive thermogenesis.

*AT*, adaptive thermogenesis; *w*_*0*_, starting weight; *w(t)*, weight at any given time.

Using the expressions above, a mathematical model of weight loss was assembled and defined as an ordinary differential equation as described by Equation 5:

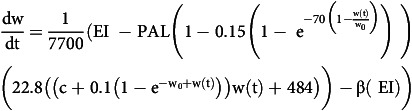




Equation 5: Original mathematical model of weight loss. 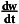 represents the rate of weight change over a given time, w_0_ is the starting weight, and w(t) is the weight at any given time. EI is energy intake in kcal/d. PAL is physical activity level, and a value of 1.6 is assumed representing a moderately active lifestyle^(14)^. This value is multiplied by REE to account for PAEE. REE is estimated using the Cunningham equation^(13)^ based on FFM, adjusted for AT. An exponential increase in AT is assumed during the weight-loss phase, persisting until a total weight loss of 10% is achieved, at which point AT stabilises at a value equivalent to 15% of REE. The parameter c represents FFM% which is estimated using gender-specific expressions developed by our group and multiplied by w(t) to produce a value for FFM (kg). An exponential increase in FFM% is assumed during the weight-loss phase, persisting until a total weight loss of 10% is achieved at which point FFM% stabilises approximately 10 percentage points higher than baseline. The parameter 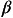 represents DIT coefficient which is assumed a value of 0.05 in an overweight/obese population^(18,19)^. This value is multiplied by EI to account for DIT. An energy conversion of 7700 kcal per kg is assumed to convert energy deficit to weight loss.

### Model validation

The proposed mathematical model is tested using weight-loss data from a large retrospective database of female clients provided by LighterLife UK Ltd (LL; Essex, UK). Relevant data was extracted from the client database and anonymised by LL personnel prior to sharing with the study investigators for analysis.

**Dietary intervention:** LL is a commercial weight management company that offers a very low-energy total-diet replacement (TDR) plan. Clients consume four food packs per day (e.g. soups, shakes, pots, and meals) replacing all conventional food, providing 600–800 kcal/d, >50 g protein, 50–75 g carbohydrate, ∼17 g fat, 14 g fibre and 22–28 essential micronutrients. Following the active weight-loss phase, clients follow a standardised protocol for food reintroduction where food packs are gradually decreased and replaced with conventional foods. Clients attend weekly group meetings delivered by trained LL counsellors consisting of weigh-ins and optional behaviour support.

**Inclusion criteria:** Individuals self-referring to a very low-energy TDR programme for 6–12 weeks from the years 2017–2021 were considered eligible for inclusion in the extracted data. Clients were considered ineligible if they had previously enrolled on a LL programme and/or gained weight during the programme. Once an appropriate study population was determined, clients were anonymised by LL personnel using a client ID. The anonymised data were then disseminated to the study investigators for analysis.

**Data extraction:** Demographic and anthropometric data from LL clients enrolled in TDR programme were extracted. Interventions 6–12 weeks in duration were examined to reflect a typical TDR programme duration. The proposed mathematical model was used to predict:

1. End-weight and weight loss in LL participants enrolled on VLED programmes of different intervention lengths (6–12 weeks).
2. End-weight and weight loss at 7-d intervals in a subset of LL participants with sequential weight measures.

Mean error, that is, the mean difference between actual and predicted end-weight expressed as a percentage of actual end-weight, was determined post-intervention and at weekly intervals.

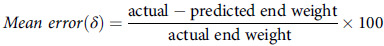




*Modelling energy intake*: In the LL cohort, a step function was used to describe EI, where a one-step increase in kcal intake was assumed from week 10 to account for those in the food reintroduction phase and due to expected deviation from dietary prescription in the later stages of the intervention as described by Equation 6:

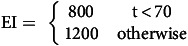




Equation 6: Step function for EI consisting of two intervals of different constant values with a jump between the horizontal line segments. For dieting durations <10 weeks, an intake of 800 kcal/d is assumed. For dieting durations >10 weeks, an intake of 1200 kcal/d is assumed. *EI*, energy intake; *t*, time.

*Statistical analysis*: Data are presented as mean and standard deviation. The Shapiro–Wilk test was used to test the normality of the data. A two-way ANOVA multiple comparisons test was performed to evaluate differences in actual weight loss vs. predicted weight loss across several timepoints. A one-way ANOVA or non-parametric Kruskal–Wallis test was performed to evaluate weekly weight changes. *P*-value <0.05 was considered as statistically significant.

## Results

A total of 983 females were included in the present analysis. Clients were on average 47 years old with a mean body weight of 93.31 ± 19.18 kg and BMI of 34.51 ± 6.52 kg/m^2^. The largest proportion of individuals enrolled in the programme for 8 weeks (n = 175), and the smallest proportion enrolled for 12 weeks (n = 84). The mean intervention length was 9 weeks.

### Prediction of post-interventional weight loss

Across all intervention lengths, an overall mean weight loss of 7.90 ± 4.63 kg was observed. On average, participants lost 8.45 ± 4.55% of their starting body weight. Our mathematical model was associated with an average mean error of 0.75 ± 5.12% equivalent to 1.07 ± 4.52 kg. The best agreement between actual and predicted end-weight was observed in those enrolled on a 6-week intervention, with a mean error of –0.28 ± 4.22% (0.16 ± 3.65 kg). Mean error increased with intervention length, with the largest mean error observed in those enrolled on a 12-week intervention (2.36 ± 6.26% equivalent to 2.45 ± 5.57 kg). Table 1 summarises the post-intervention mean error of our mathematical model across all timepoints.

**Table 1.** Post-intervention mean error of mathematical model in the LighterLife study

Weeks	n	δ
6	193	–0.28 (4.22)
7	181	0.59 (4.37)
8	196	0.40 (4.70)
9	158	0.47 (4.93)
10	155	1.09 (6.03)
11	133	1.75 (5.72)
12	93	2.36 (6.26)
Overall	1109	0.75 (5.12)

Data presented as mean (SD). Participants followed a very low-energy total-diet replacement intervention for 6–12 weeks providing 600–800 kcal/d. End-weight was predicted by our mathematical model and compared to that observed in LighterLife participants. δ, mean error ([actual-predicted end-weight/actual end-weight] × 100).

### Prediction of weekly weight loss

Total weight loss increased with dieting duration (r = 0.96) ranging from 2.28 ± 1.56 kg at week 1 to 9.55 ± 4.92 at week 11. In contrast, a negative correlation was observed between the rate of weight loss and dieting duration (r = 0.75), decreasing from 2.28 ± 1.56 kg/week at week 1 to 0.74 ± 5.13 kg/week at week 12. Absolute weight loss (*P* <0.0001) and rate of weight loss (*P* < 0.0001) changed significantly over time as determined by one-way ANOVA.

**Fig. 1.**
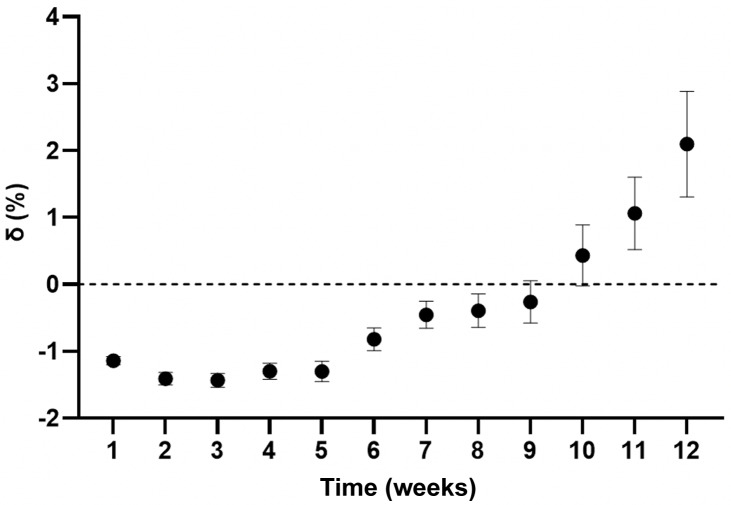
Mean error of mathematical model at weekly intervals. Data presented as mean (SEM). End-weight in LighterLife participants was predicted by our mathematical model and compared to that observed. Week 1, –1.14 ± 1.68%, n = 713; week 2, –1.41 ± 2.4%, n = 656; week 3, –1.44 ± 2.5%, n = 606; week 4, –1.30 ± 2.93%, n = 599; week 5, –1.30 ± 3.64%, n = 565; week 6, –1.82 ± 4.09%, n = 578; week 7, –0.46 ± 4.4%, n = 465; week 8, –0.40 ± 4.91%, n = 388; week 9, –0.26 ± 5.27%, n = 277; week 10, 0.43 ± 6.28%, n = 190; week 11, 1.06 ± 5.96%, n = 121; week 12, 2.10 ± 6.07%, n = 59. δ, mean error ([actual-predicted end-weight/actual end-weight] × 100).

Figure 1 summarises the mean error of our model at weekly intervals for 12 weeks ranging from –1.44 ± 2.52% (–1.13 ± 2.14 kg) at week 3 to 2.09 ± 6.07% (2.24 ± 5.39 kg) at week 12. The best agreement was observed in 7–9-week interventions, predicting end-weight within 0.50% (<0.50 kg) of that observed.

When translated into predicted weight loss, our model underestimated weight loss in the first 7 weeks of the intervention and overestimated weight loss from week 8 onwards (Table 2).

**Table 2.** Actual versus predicted weight loss at weekly intervals in females enrolled on the LighterLife study

Week	*n*	Predicted weight loss (kg)	Actual weight loss (kg)	Difference (kg)	*P*-value
1	713	1.29 (0.42)	2.28 (1.56)	–0.99 (1.49)	<0.001
2	656	2.55 (0.84)	3.70 (2.03)	–1.15 (1.97)	<0.001
3	606	3.60 (1.20)	4.73 (2.31)	–1.13 (2.14)	<0.001
4	599	4.64 (1.59)	5.58 (2.65)	–0.95 (2.54)	<0.001
5	565	5.61 (1.97)	6.46 (3.17)	–0.85 (3.09)	<0.001
6	578	6.64 (2.38)	6.97 (3.43)	–0.34 (3.52)	ns
7	465	7.56 (2.64)	7.57 (3.73)	–0.01 (3.81)	ns
8	388	8.44 (3.09)	8.34 (4.26)	0.10 (4.20)	ns
9	277	9.64 (3.39)	9.39 (4.63)	0.24 (4.57)	ns
10	190	10.41 (3.63)	9.47 (5.52)	0.93 (5.41)	0.0092
11	121	10.97 (3.43)	9.55 (4.92)	1.42 (5.12)	0.0006
12	59	11.18 (3.85)	8.94 (5.13)	2.24 (5.39)	<0.001

Data presented as mean (SD). Participants followed a very low-energy total-diet replacement intervention for 6–12 weeks providing 600–800 kcal/d. Weekly weight loss in LighterLife participants was predicted by our mathematical model and compared to that observed. The statistical significance of differences between actual and predicted weight loss was assessed using paired *t*-tests.

**Fig. 2.**
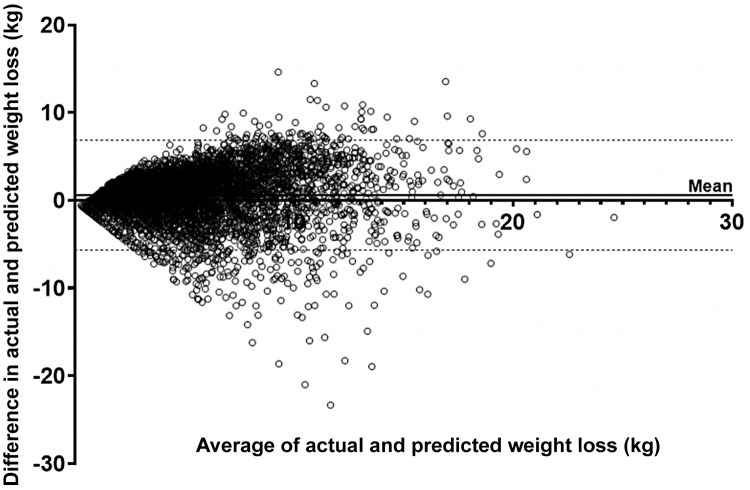
Bland–Altman analysis to investigate the agreement between actual and predicted weight loss. Participants followed a very low-energy total-diet replacement intervention for 6–12 weeks providing 600–800 kcal/d. Average = (actual weight loss + predicted weight loss)/2. The dotted lines represent the upper and lower limits of agreement ( ± 2 SD). The solid line represents the average difference (kg).

Actual weight loss can be compared with predicted weight loss using the Bland and Altman method as illustrated in Fig. 2. Mean bias between actual weight loss and weight loss predicted by mathematical modelling was 0.61 ± 3.19 kg. The 95% confidence interval for the difference was –5.65–6.86 kg.

## Discussion

In the present study, a proposed mathematical predicted post-intervention end-weight within 0.75% of that observed in females enrolled on a 6–12 weeks TDR intervention, reflecting a mean weight-loss overestimation of 1.07 kg. Mean error increased with intervention length, from –0.28%, equivalent to 0.16 kg in 6-week interventions, to 2.36% equivalent to 2.45 kg in 12-week interventions.

The extraction of weekly weights allowed us to determine where our mathematical model was most and least accurate. Findings showed that our model underestimated weight loss in the first 6 weeks of the intervention, particularly in the first week where weight loss was 75% greater than that predicted. This is likely due to the rapid depletion of glycogen pools and associated water and electrolytes (stored in a proportion of 1:3 g, respectively) in the first 7–10 d equivalent to a total weight loss of 1.5–2.0 kg^(1,20–22)^.

Alternatively, mathematical modelling overestimated weight loss beyond week 8, possibly due to relaxed compliance in those enrolled on the programme for longer durations.

While data on the number of TDR products purchased per week were provided, this may not necessarily equate to TDR products consumed, particularly in participants enrolled on the intervention for over 8 weeks who may be using partial meal replacement or transitioning to a weight maintenance phase. Furthermore, the frequency of diet relapse is likely to increase with intervention length, particularly in those not participating in a gradual food reintroduction process, where rapid carbohydrate refeeding can result in a significant surge in hunger hormones and appetite. It is noteworthy that only 8.5% of the study population enrolled on the programme for 12 weeks, meaning relaxed compliance in one given participant will significantly impact mean weight loss.

Overall, our model was associated with a mean error of 0.60%, reflecting an average weight-loss overestimation of 0.27 kg. The best agreement between actual and predicted weight loss was observed from weeks 6–9 (ns *p* > 0.3827), representing the intervention length of ∼48% of study participants, where predicted end-weight was within 0.30% (0.35 kg) of the observed. Furthermore, this range encompasses the mean intervention length of 8.7 weeks suggesting our model can produce intervention end-weight with reasonable accuracy. Despite this, our weekly analysis identifies points of the weight-loss phase where our model requires further refinement.

Based on present findings, the following refinements were applied to our mathematical model: (i) new predictor equation for REE: REE is estimated using the Mifflin equation based on FFM^(23)^, developed in a population with both lean and obese individuals, thus more reflective of our study population, and (ii) new formulation for FFM: FFM is modelled as a function of weight loss. A linear increase in FFM% is assumed during the weight-loss phase, persisting until a total weight loss of 20% at which point FFM% stabilises approximately 10 percentage points higher than baseline, as described by Equation 7:

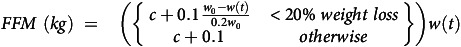




Equation 7: Refined expression for FFM change.

*FFM*, fat-free mass; *c*, baseline fat-free mass (%); *w*_*0*_, starting weight; *w(t)*, weight at any given time.

### Strengths and limitations

The proposed model requires only simple inputs of EI and PAL and thus can be used without expensive lab equipment. While several mathematical weight-loss models do exist, few rely only on clinically available anthropometric and demographic variables that are readily available in a clinical environment. Such a model may be used for (i) prescribing dietary intake in terms of energy deficit, (ii) setting target weights and timescales, (iii) monitoring dietary compliance, and (iv) patient/client motivation and self-management.

A key strength of the study is a large sample size strengthening the validity and reliability of our results. Individuals are self-referring and self-funding clients and thus are likely to be motivated and may exhibit higher levels of adherence.

The present study represents the nature of weight loss in free-living individuals, rather than study participants participating in a tightly regulated clinical trial. As such, the proposed mathematical model can be used to identify those deviating from the expected weight-loss trajectory who may require additional input. However, the accuracy of the proposed model is limited by the reliance on assumptions regarding EI and EE implemented to improve model applicability.

To quantify one side of the EB equation, that is, EE, it is important to keep the other side (EI) is as controlled as possible. Thus, to minimise potential error, our model is validated using weight-loss data from a TDR programme, using specially formulated food products of known energy content. If used as directed, TDR products are an accurate method of estimating EI.

However, it should be considered that there is likely much less variation in EI using TDR products than would be using food-based weight-loss programmes. Thus, it remains unclear if similar accuracy can be observed in food-based dietary interventions.

Predictor equations are developed using linear regression analysis which assumes a proportional increase in REE with increasing body weight. However, in morbidly obese subjects, excess weight is predominately FM rather than metabolic tissue. Furthermore, excess FFM is predominantly low-metabolic skeletal muscle rather than high-metabolic organs, for example, the brain, liver, and kidney^(24,25)^. Thus, REE increases at a slower rate in a more curvilinear manner^(26–28)^ rather than a linear increase as assumed by most predictive equations^(24)^.

Our model includes a term for AT based on observations from clinical weight-loss studies. While longitudinal studies support the existence of AT, significant between-individual variance is observed^(3)^. AT is suggested to be biologically determined, whereby two existing phenotypes (‘thrifty’ and ‘spendthrift’) differ in their capacities to regulate EE in response to altered energy availability^(7,29)^. Subsequently, it should be considered that the term for AT assumed by our model may not be representative of all study participants. The proposed model assumes an immediate adaptive response to energy deficit. However, the onset time for AT is a matter of debate with a considerable amount of research suggesting AT takes weeks to develop^(15)^.

Finally, the proposed model relies on the assumption that those purchasing over twenty-eight products per week are following the prescribed four products per day. Additional intake from conventional foods was not recorded and therefore our model relies on assumptions regarding programme adherence. Thus, despite the prescriptive nature of TDR, true values for EI remain uncertain.

### Conclusion

Mathematical models have been used to simulate EB and weight dynamics for decades, yet application in clinical weight loss remains limited. The model proposed in the present study estimates energy deficit by simulating dynamic changes in EE using simple baseline parameters of gender, weight, and EI. In a large cohort of weight-reducing females, the proposed model was associated with a mean bias of 0.61 kg. Limitations of model assumptions need to be examined before clinical application; however, there is no doubt that mathematical modelling has a valuable place in the treatment of overweight and obesity.

## References

[1] Buchholz AC , Schoeller DA. Is a calorie a calorie? Am J Clin Nutr. 2004;79:899S–906S. 15113737 10.1093/ajcn/79.5.899S

[2] Thomas DM , Gonzalez MC , Pereira AZ , et al. Time to correctly predict the amount of weight loss with dieting. J Acad Nutr Diet. 2014;114:857–861. 24699137 10.1016/j.jand.2014.02.003 PMC4035446

[3] Hall KD , Guo J. Obesity energetics: Body weight regulation and the effects of diet composition. Gastroenterology. 2017;152:1718–1727. 28193517 10.1053/j.gastro.2017.01.052 PMC5568065

[4] Gallagher D , Kelley DE , Thornton J , et al. MRI Ancillary Study Group of the look AHEAD research group. Changes in skeletal muscle and organ size after a weight-loss intervention in overweight and obese type 2 diabetic patients. Am J Clin Nutr. 2017;105:78–84. 27881389 10.3945/ajcn.116.139188 PMC5183727

[5] Casanova N , Beaulieu K , Finlayson G , et al. Metabolic adaptations during negative energy balance and their potential impact on appetite and food intake. Proc Nutr Soc. 2019;78:279–289. 30777142 10.1017/S0029665118002811

[6] Nunes CL , Casanova N , Francisco R , et al. Does adaptive thermogenesis occur after weight loss in adults? A systematic review. Br J Nutr 2022;127:451–469. 33762040 10.1017/S0007114521001094

[7] Muller MJ , Enderle J & Bosy-Westphal A. Changes in energy expenditure with weight gain and weight loss in humans. Curr Obes Rep. 2016;5:413–423. 27739007 10.1007/s13679-016-0237-4 PMC5097076

[8] Muller MJ , Enderle J , Pourhassan M , et al. Metabolic adaptation to caloric restriction and subsequent refeeding: the Minnesota Starvation Experiment revisited. Am J Clin Nutr 2015;102:807–819. 26399868 10.3945/ajcn.115.109173

[9] Dulloo AG , Jacquet J. Adaptive reduction in basal metabolic rate in response to food deprivation in humans: a role for feedback signals from fat stores. Am J Clin Nutr. 1998;68:599–606. 9734736 10.1093/ajcn/68.3.599

[10] Thomas DM , Martin CK , Lettieri S , et al. Can a weight loss of one pound a week be achieved with a 3500-kcal deficit? Commentary on a commonly accepted rule. Int J Obes. 2013;37:1611–1613. 10.1038/ijo.2013.51 PMC4024447 23628852

[11] Hall KD , Sacks G , Chandramohan D , et al. Quantification of the effect of energy imbalance on body weight. Lancet 2011;378:826–837. 21872751 10.1016/S0140-6736(11)60812-X PMC3880593

[12] Thomas DM , Scioletti M & Heymsfield SB. Predictive mathematical models of weight loss. Curr Diab Rep. 2019;19:93. 31473830 10.1007/s11892-019-1207-5

[13] Cunningham JJ. A reanalysis of the factors influencing basal metabolic rate in normal adults. Am J Clin Nutr. 1980;33:372–374. 10.1093/ajcn/33.11.2372 7435418

[14] Black AE , Coward WA , Cole TJ , et al. Human energy expenditure in affluent societies: analysis of 574 doubly labelled water measurements. Eur J Clin Nutr 1996;50:72–92. 8641250

[15] Egan A , Collins A. Dynamic changes in energy expenditure in response to underfeeding: a review. Proc Nutr Soc 2022;81:199–212. 35103583 10.1017/S0029665121003669

[16] Leibel R , Rosenbaum M , Hirsch J. Changes in energy expenditure resulting from altered body weight. N Eng J Med. 1995;332:621–628. 10.1056/NEJM199503093321001 7632212

[17] Weigle D , Sande K , Iverius P , Monsen E , Brunzell J. Weight loss leads to a marked decrease in nonresting energy expenditure in ambulatory human subjects. Metabolism. 1988;37:930–936. 3173112 10.1016/0026-0495(88)90149-7

[18] Wang C , Strouse S , Saunders A. Studies on the metabolism of obesity: III. The specific dynamic action of food. Arch Intern Med. 1924;34:573–583.

[19] De Jonge L , Bray G. The thermic effect of food and obesity: a critical review. Obes Res. 1997;35:622–631. 10.1002/j.1550-8528.1997.tb00584.x 9449148

[20] Heymsfield SB , Thomas D , Nguyen AM , et al. Voluntary weight loss: a systematic review of early phase body composition changes. Obes Rev. 2011;12:e348–e361. 20524998 10.1111/j.1467-789X.2010.00767.x

[21] Ashtary-Larky D , Ghanavati M , Lamuchi-Deli N , et al. Rapid weight loss vs. slow weight loss: which is more effective on body composition and metabolic risk factors? Int J Endocrinol Metab. 2017;15:e13249. 29201070 10.5812/ijem.13249 PMC5702468

[22] Heinitz S , Hollstein T , Ando T , et al. Early adaptive thermogenesis is a determinant of weight loss after six weeks of caloric restriction in overweight subjects. Metabolism. 2020;110:154303. 32599082 10.1016/j.metabol.2020.154303 PMC7484122

[23] Mifflin MD , St Jeor ST , Hill LA , et al. A new predictive equation for resting energy expenditure in healthy individuals. Am J Clin Nutr 1990;51:241–247. 2305711 10.1093/ajcn/51.2.241

[24] Madden AM , Mulrooney HM , Shah S. Estimation of energy expenditure using prediction equations in overweight and obese adults: a systematic review. J Hum Nutr Diet. 2016;29:458–476. 26923904 10.1111/jhn.12355

[25] Heymsfield SB , Thomas DM , Bosy-Westphal A , et al. The anatomy of resting energy expenditure: body composition mechanisms. Eur J Clin Nutr 2019;73:166–171. 30254244 10.1038/s41430-018-0319-3 PMC6410366

[26] Forbes GB , Brown MR , Welle SL , et al. Deliberate overfeeding in women and men: energy cost and composition of the weight gain. Br J Nutr 1986;56:1–9. 3479191 10.1079/bjn19860080

[27] Horgan GW , Stubbs J. Predicting basal metabolic rate in the obese is difficult. Eur J Clin Nutr. 2003;57:335–340. 12571669 10.1038/sj.ejcn.1601542

[28] Bosy-Westphal A , Eichhorn C , Kutzner D , et al. The age-related decline in resting energy expenditure in humans is due to the loss of fat-free mass and to alterations in its metabolically active components. J Nutr. 2003;133:2356–2362. 12840206 10.1093/jn/133.7.2356

[29] Reinhardt M , Thearle MS , Ibrahim M , et al. A Human Thrifty Phenotype Associated With Less Weight Loss During Caloric Restriction. Diabetes. 2015;64:2859–2867. 25964395 10.2337/db14-1881 PMC4512223

